# Mechanical ventilation in patients with acute ischemic stroke: from pathophysiology to clinical practice

**DOI:** 10.1186/s13054-020-2806-x

**Published:** 2020-04-07

**Authors:** Jiguo Gao, Chunkui Zhou, Hongliang Zhang

**Affiliations:** 1 Department of Neurology, First Hospital of Jilin University, Changchun, China; 2 Department of Life Sciences, National Natural Science Foundation of China, Shuangqing Road 83#, Beijing, 100085 China

Robba and colleagues are to be commended for exploring the pathophysiology of brain-lung interactions after acute ischemic stroke and the management of mechanical ventilation in these patients via a narrative review [1]. Pulmonary complications are common in patients with acute ischemic stroke and are associated with a high risk of mortality. The mechanism of lung damage after brain injury is described as a “double-hit model,” which appears adequate to explain some pathophysiological phenomena in the lung secondary to stroke [1]. However, pulmonary complications after acute ischemic stroke may occur with more complex mechanism.

Acute respiratory infection usually of bacterial origin, particularly in the week preceding stroke, is a significant risk factor for cerebral infarction; acute infection acts as a trigger for acute stroke [2]. In this regard, the possibility cannot be excluded that some of the infections reported early poststroke actually developed prior to stroke onset but worsened thereafter [2].

We agree that mechanisms other than dysphagia or compromised level of consciousness are involved in stroke-associated pneumonia [1]. However, both immune activation and immune immunodepression occur after acute ischemic stroke. Normally well-balanced brain-immune interactions may become dysregulated [2]. Experimental and clinical data support that stroke may impair immunity or induce immunodepression [2]. Factually, the PREDICT study confirmed that stroke-induced immunodepression syndrome is an independent risk factor for stroke-associated pneumonia, instead of aspiration pneumonia [3].

Acute ischemic brain injury swiftly activates the sympathetic, parasympathetic, and HPA axis pathways, leading to the release of norepinephrine (NE), acetylcholine, and glucocorticoids (GCs). Adrenergic and the HPA axis pathways act synergistically to induce splenic atrophy and natural killer (NK) cell deficiency in the periphery via coordinated effects of NE and GCs [4]. Moreover, ischemic stroke may cause a significant increase in bronchoalveolar lavage fluid macrophages and neutrophils and whole lung tissue pro-inflammatory IL-1β mRNA expression in experimental mice [5]. IL-1β is known to be involved in the development of acute lung injury (ALI) and/or acute respiratory distress syndrome and has been shown to be one of the most biologically active cytokines in ALI [5].

In summary, pulmonary complications are associated with a high risk of mortality in patients with acute ischemic stroke. The mechanism of lung damage after brain injury merits further investigation.

## Authors’ response

Denise Battaglini, Giulia Bonatti, Chiara Robba, Patricia RM Rocco, Paolo Pelosi

**Fig. 1:**
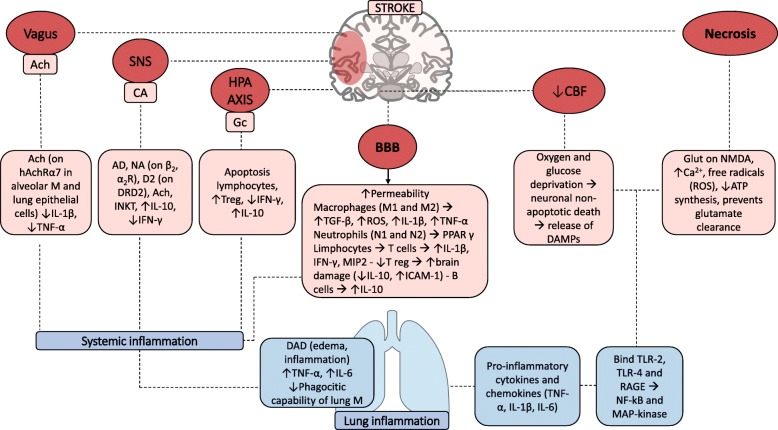
Inflammatory cascade after acute ischemic stroke. This figure represents the complex inflammatory cascade activated after acute ischemic stroke. *Vagus* efferences, sympathetic nervous system and hypothalamic-pituitary axis enhance immunosuppression by releasing Ach, catecholamine, and glucocorticoids, respectively. These molecules bind their specific receptors (nAchRα7 for Ach; β_2_, α_2_, and D2 for catecholamines) inducing apoptosis of lymphocytes, increasing the release of IL-10, and decreasing the release of IFN-γ, TNF-α, and IL-1β. At the same time, the reduction of cerebral blood flow caused by vessel’s ischemia determines oxygen and glucose deprivation with consequent neuronal and glial death and release of DAMPs. DAMPs include glutamate, ATP, and ROS and bind TLR-2, TLR-4, and RAGE thus inducing NF-kB and MAP kinase activation. Moreover, the blood-brain barrier damage with increased permeability yields immune cell translocation and enhanced inflammatory response. The peripheral effect on the lung includes diffuse alveolar damage with edema and inflammation and increases TNF-α and IL-6 levels, also reducing phagocytic capability of macrophages. Ach, acetylcholine; SNS, sympathetic nervous system; HPA, hypothalamic-pituitary-adrenal axis; BBB, blood-brain barrier; CBF, cerebral blood flow; M, macrophages; IL, interleukin; Gc, glucocorticoids; TNF, tumor necrosis factor; Treg, T regulator; IFN, interferon; TGF, transforming growth factor; ROS, radicals of oxygen; PPAR, peroxisome proliferator-activated receptor; MIP, macrophage inflammatory protein; ICAM, intracellular adhesion molecule; DAMPS, damage-associated molecular patterns; ATP, adenosine triphosphate; TLR, toll-like receptor; DAD, diffuse alveolar damage; RAGE, advanced glycation end products, DRD2, dopamine receptor D2; INKT, invariant natural killer

Dear Editor,

We would like to thank Zhang and collaborators for reading with interest our recent review [1] concerning the role of mechanical ventilation in patients with acute ischemic stroke. The authors highlighted the role of acute ischemic stroke on pulmonary complications and their association with clinical outcome and death. Indeed, the mechanisms associated with pulmonary complications after acute ischemic stroke are complex and further studies are required. In Fig. 1, we highlighted the main mechanisms of acute ischemic stroke-induced lung inflammation.

After vessel occlusion, oxygen and glucose deprivation induces neuronal and glial cell death, followed by the release of damage-associated molecular patterns (DAMPs) that include adenosine triphosphate (ATP), glutamate, and reactive oxygen species (ROS). These molecules bind to toll-like receptors (TLRs)-2, TLR-4, and receptor for advanced glycation end products (RAGE), activating resident microglial cells and stimulating the release of pro-inflammatory cytokines (such as tumor necrosis factor (TNF)-α, interleukin (IL)-1β, and IL-6). Glutamate binds *N*-methyl-d-aspartate receptors (NMDA) increasing calcium influx and producing ROS [6].

Sympathetic nervous system, efferent *vagus* nerve, and hypothalamic-pituitary-adrenal (HPA) axis are overactivated by acute ischemic stroke and may induce immunosuppression. Particularly, the efferent *vagus* acts through the release of acetylcholine (Ach) that binds nicotinic acetylcholine receptor (nAchRα7) both on lung alveolar macrophages and epithelial cells reducing inflammation and neuronal cells protecting against oxidative stress [7]. Catecholamines (on β_2_, α_2_, and D2 receptors) are released by sympathetic nervous system and activate T regulator lymphocytes with the liberation of interferon (IFN)-γ and IL-10. Moreover, HPA axis induces glucocorticoid secretion, thus activating apoptosis of lymphocytes, releasing IL-10, and reducing IFN-γ liberation by T cells [7].

Blood-brain barrier permeability is increased, and inflammatory cells are released into the systemic circulation. Microglia (M) are polarized into M1 (classically activated) or M2 (alternatively activated) phenotypes. Activation of these phenotypes affects the prognosis of stroke [8]. The M2 phenotype plays a protective role in the brain by releasing transforming growth factor-β, whereas inflammatory cytokines induced by M1 phenotype aggravate brain injury after stroke [8].

Therefore, systemic inflammation is activated. Diffuse alveolar damage with lung edema and inflammation have been observed after acute ischemic stroke in mice associated with enhanced pro-inflammatory cytokines and reduced phagocytic capability of lung macrophages [6].

Although significant progress has been made in the pathogenesis of stroke and its interaction with peripheral organs, further investigations are needed to elucidate stroke-induced immunosuppression and its potential therapeutic strategies.

## Data Availability

Not applicable.
